# Rapid sequencing of MRSA direct from clinical plates in a routine microbiology laboratory

**DOI:** 10.1093/jac/dkz170

**Published:** 2019-04-30

**Authors:** Beth Blane, Kathy E Raven, Danielle Leek, Nicholas Brown, Julian Parkhill, Sharon J Peacock

**Affiliations:** 1 Department of Medicine, University of Cambridge, Box 157 Addenbrooke’s Hospital, Hills Road, Cambridge CB2 0QQ, UK; 2 Clinical Microbiology and Public Health Laboratory, Public Health England, Cambridge CB2 0QQ, UK; 3 Wellcome Sanger Institute, Wellcome Genome Campus, Hinxton, Cambridge CB10 1SA, UK; 4 London School of Hygiene & Tropical Medicine, Keppel Street, London WC1E 7HT, UK

## Abstract

**Background:**

Routine sequencing of MRSA could bring about significant improvements to outbreak detection and investigation. Sequencing is commonly performed using DNA extracted from a pure culture, but overcoming the delay associated with this step could reduce the time to infection control interventions.

**Objectives:**

To develop and evaluate rapid sequencing of MRSA using primary clinical cultures.

**Methods:**

Patients with samples submitted to the clinical laboratory at the Cambridge University Hospitals NHS Foundation Trust from which MRSA was isolated were identified, the routine laboratory culture plates obtained and DNA extraction and sequencing performed.

**Results:**

An evaluation of routine MRSA cultures from 30 patients demonstrated that direct sequencing from bacterial colonies picked from four different culture media was feasible. The 30 clinical MRSA isolates were sequenced on the day of plate retrieval over five runs and passed quality control metrics for sequencing depth and coverage. The maximum contamination detected using Kraken was 1.09% fragments, which were identified as *Prevotella dentalis*. The most common contaminants were other staphylococcal species (25 isolate sequences) and *Burkholderia dolosa* (11 isolate sequences). Core genome pairwise SNP analysis to identify clusters based on isolates that were ≤50 SNPs different was used to triage cases for further investigation. This identified three clusters, but more detailed genomic and epidemiological evaluation excluded an acute outbreak.

**Conclusions:**

Rapid sequencing of MRSA from clinical culture plates is feasible and reduces the delay associated with purity culture prior to DNA extraction.

## Introduction

Numerous studies have confirmed that bacterial sequencing provides much greater discrimination between isolates of the same lineage than previous typing methods, and could bring about significant improvements in outbreak investigation.^1–6^ This has led to calls for prospective sequencing of key nosocomial pathogens such as MRSA to detect outbreaks in real time.^7^ Although the case for routine bacterial sequencing to augment hospital outbreak detection is growing, several barriers need to be overcome before information is generated with sufficient speed to influence infection control practice. One of these is to remove the reliance on pure bacterial culture for DNA extraction, which requires sub-culture from the primary clinical culture plate and can introduce a delay of up to 1 working day. This could be overcome by performing sequencing of material extracted from a bacterial colony on the first culture plate to become positive from a clinical sample.

Methodology for rapid single-colony WGS has been developed and applied in a research setting, in which 17 bacterial pathogens responsible for severe human infection were grown using standard diagnostic media and incubation conditions and successfully sequenced.^8^ This suggests that colony pick may be feasible in routine practice but has not been tested using clinical culture plates in a routine laboratory. These cultures are more challenging since they may contain multiple bacterial species, have a low growth of the bacterial species of interest, and may have few or no single identifiable colonies. Here, we evaluated the feasibility of rapid WGS of MRSA from clinical culture plates.

## Materials and methods

### Study setting, patients and sample identification

The study was conducted at the Clinical Microbiology and Public Health Laboratory at the Cambridge University Hospitals NHS Foundation Trust (CUH), UK, under ethics approval from the National Research Ethics Service (reference 11/EE/0499) and the Cambridge University Hospitals NHS Foundation Trust Research and Development Department (reference A092428). Consecutive patients with samples that grew MRSA were identified every day for 7 days in April 2018. Putative or confirmed MRSA-positive samples were flagged by laboratory staff and any available culture plates were retrieved on the same day. Samples were deduplicated so that each patient only had a single MRSA isolate included in the study. Patients and samples were renumbered with an anonymous study code. Information was recorded on date and place of sampling, sample type (screen or clinical sample), ward movement (if an inpatient), general practitioner (GP) and residential postcode. Epidemiological evaluation of patients with genetically related MRSA assumed that shared ward (excluding accident and emergency), postcodes or GP surgeries represented strong epidemiological links, as described previously.^9^

### Bacterial identification and colony pick

Putative *Staphylococcus aureus* on clinical plates were confirmed using the Staph Latex Kit (Pro-Lab Diagnostics). A single 2–3 mm colony was picked for DNA extraction when present, which is roughly the colony size after overnight incubation on Columbia blood agar (CBA) or chocolate blood agar plates. Where colonies were smaller than 2 mm, which was common on *Brilliance* MRSA or Mueller–Hinton agar plates, several colonies were picked using a 1 μL loop. Where bacterial growth was confluent, a 1 μL loopful was taken. If there were several positive plates for one clinical sample, the plate with the least visible background contamination was selected. The loopful of bacteria was agitated in 174 μL of PBS to create a bacterial suspension, after which the same loop was used to create a purity CBA plate for bacterial storage after overnight incubation. Isolates were stored at −80°C in Microbank vials (Pro-Lab Diagnostics) until further use.

### Sequencing and data analysis

DNA was extracted from the colony suspension using the QIAgen Mini DNA extraction kit. Sequencing libraries were made on the same day as DNA extraction using the Illumina Nextera DNA Flex Kit. Post-extraction DNA quantification and qualification were not performed, as normalization is expected using the library preparation kit. Library quantification was performed using the Qubit 4 fluorometer (ThermoFisher Scientific). Libraries were sequenced on an Illumina MiniSeq with a run time of 13 h using the high-output 150 cycle MiniSeq cartridge and the Generate Fastq workflow and paired-end reads. Data were saved to an external hard drive prior to analysis. Additional non-study MRSA isolates (not reported here) were sequenced in parallel with the study MRSA isolates to increase cost efficiency. For each of the five runs performed, we sequenced 21 clinical *S. aureus* isolates plus three controls [a positive control (MRSA MPROS0386), a negative control (*E. coli* NCTC12241) and a no-template control]. The controls passed internal quality control (QC) metrics on all runs. Sequence data for the study MRSA are available from the European Nucleotide Archive (https://www.ebi.ac.uk/ena) under the accession numbers listed in Table S1 (available as Supplementary Data at *JAC* Online).

Species identification was performed using Kraken version 1 (https://ccb.jhu.edu/software/kraken/) with the miniKraken database (https://ccb.jhu.edu/software/kraken/dl/minikraken_20171019_8GB.tgz). ST was identified using Ariba (https://github.com/sanger-pathogens/ariba/wiki/MLST-calling-with-ARIBA). The presence of *mecA* (accession number HE681097, position 2790560:2792566) was identified using Ariba (https://github.com/sanger-pathogens/ariba). Fastq files were mapped to clonal complex-specific references using SMALT for all isolates that were assigned to an ST that contained two or more clinical isolates (https://www.sanger.ac.uk/science/tools/smalt-0). Clonal complex (CC) references were as follows: CC1, MW2 (accession number BA000033); CC5, N315 (BA000018); CC8, USA300 (CP000255.1); CC22, HO 5096 0412 (HE681097); CC45, CA347 (CP006044); and CC59, M013 (CP003166). Mobile genetic elements were removed using the files available at https://figshare.com/authors/Francesc_Coll/5727779 and the script available in Github (https://github.com/sanger-pathogens/remove_blocks_from_aln). SNPs were identified using the script available in Github (https://github.com/sanger-pathogens/snp-sites). All clinical MRSA sequences passed internal quality metrics (confirmed as *S. aureus*, *mec* gene detected, ST assigned, 20× minimum mean depth of coverage, 80% minimum of the reference genome mapped), as described previously.^10^

## Results and discussion

We identified 33 consecutive MRSA-positive individuals over a consecutive 7 day period in April 2018. Daily collection of culture plates led to the retrieval of at least one culture from 30/33 (91%) cases. Nine samples were submitted by nine GP surgeries and the remainder were submitted by two hospitals. Twenty samples were multisite MRSA screens and 10 were diagnostic specimens (9 surface swabs and 1 intraoperative swab).

**Figure 1.**
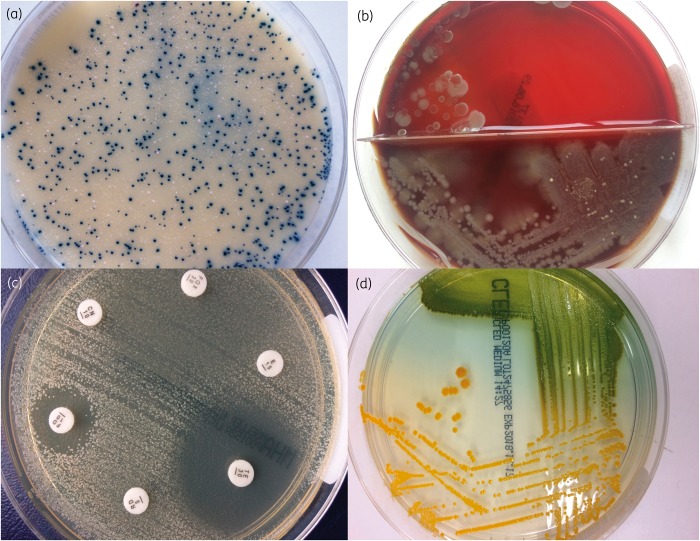
Examples of agar plates used for colony pick sequencing. (a) *Brilliance* MRSA plate. The dark blue colonies of MRSA are smaller than typically seen on blood agar plates and intermixed with white colonies that are consistent with members of the normal skin microbiota. (b) Blood/CAP agar plate. The bottom half is blood agar, which allows most bacterial species to grow. The colonies observed are consistent with coliforms and overgrowing any other bacterial species present. The top half is selective CAP agar, suppressing the growth of Gram-negative bacteria and allowing growth of *S. aureus*. (c) Mueller–Hinton agar plate used for susceptibility testing. A semi-confluent lawn is observed, from which several colonies were picked using a 1 μL loop. (d) CLED agar plate, which is used to suppress the growth of *Proteus* spp. if present and allows colonies of *S. aureus* to be picked.

Two-thirds (21/30) of cases had Mueller–Hinton agar/disc diffusion susceptibility testing plates available, which had been inoculated with a single colony picked from the primary culture plate and so were predicted to represent pure cultures. A 1 μL loop sweep was taken from each of these plates. The remaining nine cultures were primary culture plates that were highly contaminated with other bacterial species. These were *Brilliance* MRSA plates (four cases) used for multisite MRSA screens; colistin and aztreonam (CAP) plates (Oxoid, Basingstoke), which contain colistin and aztreonam to suppress the growth of Gram-negative bacteria (four cases); and a cysteine-, lactose- and electrolyte-deficient (CLED) plate (Oxoid, Basingstoke) (one case), which is used to prevent swarming of *Proteus* spp. but otherwise supports growth of most bacterial species. A single colony was picked from the CAP and CLED plates, but colonies on *Brilliance* MRSA agar were <2 mm and two or three colonies were picked from each plate. Examples of clinical plates are shown in Figure 1.

The 30 clinical MRSA isolates were successfully sequenced over five runs, with a minimum mean depth of coverage of 51.6 and a minimum of 92.1% of the reference genome mapped (Table S1). The maximum level of contamination detected was 1.09% fragments assigned to *Prevotella dentalis*. The most common contaminants were other staphylococcal species (25 isolate genomes) and *Burkholderia dolosa* (11 isolate genomes). Table S1 provides full details of species and proportion of reads associated with contamination. Isolates picked from *Brilliance* MRSA (*n *=* *4), Mueller–Hinton (*n *=* *21) and CLED plates (*n *=* *1) had similar levels of contamination (median of three, three and two species and 0.06%, 0.03% and 0.02% contamination, respectively), which were generally lower than the contamination found in isolates from CBA/CAP plates (*n *=* *4) (median 3.5 species, 0.12%).

The 30 MRSA genomes were assigned to 12 different STs (Table S1), including a novel ST (ST5142, a single-locus variant of ST59 with a single mutation in *aroE*). The most common STs were ST22 (*n *=* *8/30, 27%) and ST1 (*n *=* *5/30, 17%). Pairwise SNP analyses based on the core genome were determined and isolate clusters based on ≤50 SNPs different were used to triage cases for more detailed genomic and epidemiological analysis, as described previously.^9^ This assigned 8 isolates to three clusters, the remaining 22 isolates being less related. Cluster A contained four patients/isolates that were 19–42 SNPs apart; Cluster B contained two patients/isolates 27 SNPs apart; and Cluster C contained two patients/isolates 7 SNPs apart. The four patients in Cluster A had samples submitted from four different locations (hospital outpatient, hospital inpatient, two different GP practices). Two cases had been inpatients on the same ward 42 days apart and 5 months prior to the MRSA isolates collected during this study, with no other links identified. The two patients in Cluster B had multisite screens taken 5 days apart in the same hospital but different wards. Both had attended the same clinic on multiple occasions in the last 12 months, but no visits had overlapping dates. The two patients in Cluster C had wound swabs submitted from different locations (hospital ward and GP surgery). These cases had shared the same ward at different times (57 days apart), 4 months prior to the positive samples collected during this study. We concluded that none of these clusters constituted outbreaks associated with identifiable direct contact.

Our study indicates that colony pick sequence data were of sufficient quality to pass QC metrics, were not impeded by excessive contamination and could be used in an integrated epidemiological and genomic analysis. We have now incorporated colony pick sequencing into a routine MRSA sequencing workflow in our laboratory.

## Funding

This publication presents independent research supported by the Health Innovation Challenge Fund (WT098600, HICF-T5-342), a parallel funding partnership between the Department of Health and Wellcome. This project was also funded by a grant awarded to the Wellcome Sanger Institute (098051).

## Transparency declarations

S. J. P. and J. P. are consultants to Next Gen Diagnostics. All other authors: none to declare.

## Disclaimer

The views expressed in this publication are those of the author(s) and not necessarily those of the Department of Health or Wellcome Trust.

## Supplementary Material

dkz170_Supplementary_Data
