# Supplementary material for: Rapid sequencing of MRSA direct from clinical plates in a routine microbiology laboratory
Source: J Antimicrob Chemother. 2019 Apr 30;74(8):2153–6. doi: 10.1093/jac/dkz170 (PMC6640301; doi:10.1093/jac/dkz170)
Supplement: dkz170_Supplementary_Data [file dkz170_supplementary_data.docx]

Supplementary data

Table S1. Isolate details

| **Isolate number** | **Accession number** | **Plate type** | **Species** | **Number of reads** | **Percentage match to *S.aureus*** | **Average depth** | **Reference genome coverage %** | **ST** | ***mec* gene** | ***Staphylococcus epidermidis*** | ***Staphylococcus argenteus*** | ***Staphylococcus haemolyticus*** | ***Staphylcoccus lugdunensis*** | ***Staphylococcus saprophyticus*** | ***Staphylococcus carnosus*** | ***Staphylococcus equorum*** | ***Staphylococcus simulans*** | ***Staphylococcus pettenkoferi*** | ***Staphylococcus schleiferi*** | ***Entercoccus faecalis*** | ***Enterococcus faecium*** | ***Enterococcus phage*** | ***Burkholderia dolosa*** | ***Lactobacillus salivarus*** | ***Enterococcus durans*** | ***Clostridioides difficile*** | ***Prevotella dentalis*** |
| --- | --- | --- | --- | --- | --- | --- | --- | --- | --- | --- | --- | --- | --- | --- | --- | --- | --- | --- | --- | --- | --- | --- | --- | --- | --- | --- | --- |
| HICF0161 | ERS3122711 | Muller Hinton | Staphylococcus aureus | 2,830,886 | 85.72 | 73.04 | 99.6 | 22 | *mecA* |  |  |  |  |  |  |  |  |  |  |  |  |  |  |  |  |  |  |
| HICF0162 | ERS3122712 | Muller Hinton | Staphylococcus aureus | 3,131,604 | 88.7 | 78.23 | 93.3 | 1 | *mecA* | 0.01 |  |  |  |  |  |  |  |  |  | 0.01 |  |  |  |  |  |  |  |
| HICF0163 | ERS3122713 | Muller Hinton | Staphylococcus aureus | 2,700,058 | 90.95 | 68.43 | 92.1 | 59 | *mecA* |  | 0.01 |  |  |  |  |  |  |  |  |  |  |  |  |  |  |  |  |
| HICF0164 | ERS3122714 | *Brilliance* MRSA | Staphylococcus aureus | 2,598,758 | 88.78 | 69.08 | 99.9 | 22 | *mecA* |  |  |  |  |  |  |  |  |  |  |  |  |  |  |  |  |  |  |
| HICF0165 | ERS3122715 | Muller Hinton | Staphylococcus aureus | 2,483,946 | 88.83 | 61.59 | 92.2 | 59 | *mecA* | 0.05 | 0.01 |  |  |  |  |  |  |  |  |  |  |  |  |  |  |  |  |
| HICF0166 | ERS3122716 | Muller Hinton | Staphylococcus aureus | 2,308,036 | 89.24 | 61.59 | 99.9 | 22 | *mecA* |  |  |  |  |  |  |  |  |  |  |  |  |  |  |  |  |  |  |
| HICF0168 | ERS3122717 | Muller Hinton | Staphylococcus aureus | 3,463,122 | 85.83 | 87.01 | 92.4 | 5142 | *mecA* | 0.06 | 0.01 |  |  |  |  |  |  |  |  |  |  |  | 0.01 |  |  |  |  |
| HICF0169 | ERS3122718 | Muller Hinton | Staphylococcus aureus | 3,476,992 | 84.22 | 85.19 | 93.7 | 5 | *mecA* |  | 0.01 |  |  |  |  |  |  |  |  |  |  |  |  |  |  |  |  |
| HICF0170 | ERS3122719 | Muller Hinton | Staphylococcus aureus | 3,901,918 | 80.57 | 91.78 | 93.2 | 1535 | *mecA* | 0.02 | 0.02 | 0.16 | 0.04 | 0.02 | 0.02 | 0.03 |  |  |  |  |  |  | 0.01 | 0.01 |  |  |  |
| HICF0171 | ERS3122720 | *Brilliance* MRSA | Staphylococcus aureus | 2,541,508 | 81.17 | 64.28 | 99.7 | 22 | *mecA* | 0.06 | 0.01 |  |  |  |  |  |  |  |  |  |  |  | 0.01 |  |  |  |  |
| HICF0172 | ERS3122721 | *Brilliance* MRSA | Staphylococcus aureus | 2,809,170 | 87.33 | 72.24 | 98.2 | 22 | *mecA* | 0.25 |  |  |  |  |  |  |  | 0.07 |  |  |  |  |  |  |  |  |  |
| HICF0173 | ERS3122722 | CLED | Staphylococcus aureus | 3,099,388 | 86.46 | 77.85 | 93.6 | 5 | *mecA* |  | 0.01 |  |  |  |  |  |  |  |  |  |  |  | 0.01 |  |  |  |  |
| HICF0174 | ERS3122723 | Muller Hinton | Staphylococcus aureus | 3,116,852 | 84.81 | 77.04 | 93.7 | 149 | *mecA* | 0.01 | 0.01 |  |  |  |  |  | 0.13 |  |  |  |  |  |  |  |  |  |  |
| HICF0175 | ERS3122724 | Muller Hinton | Staphylococcus aureus | 3,921,730 | 85.62 | 98.33 | 93.2 | 45 | *mecA* |  | 0.01 |  |  |  |  |  |  |  |  |  |  |  |  |  |  |  |  |
| HICF0176 | ERS3122725 | Muller Hinton | Staphylococcus aureus | 2,921,598 | 82.64 | 72.15 | 94 | 1 | *mecA* | 0.05 | 0.01 | 0.08 |  |  |  |  |  |  |  |  |  |  | 0.01 |  | 0.02 |  |  |
| HICF0177 | ERS3122726 | CBA/CAP | Staphylococcus aureus | 2,982,306 | 87.61 | 72.91 | 93.2 | 149 | *mecA* |  |  |  |  |  |  |  | 0.15 |  |  |  |  |  | 0.01 |  |  |  |  |
| HICF0178 | ERS3122727 | Muller Hinton | Staphylococcus aureus | 3,270,898 | 90.19 | 82.41 | 92.2 | 5 | *mecA* |  | 0.01 |  |  |  |  |  |  |  |  |  |  |  |  |  |  |  |  |
| HICF0179 | ERS3122728 | CBA/CAP | Staphylococcus aureus | 2,637,988 | 87.68 | 65.37 | 92.2 | 59 | *mecA* | 0.05 | 0.01 |  |  |  |  |  |  |  |  |  |  |  | 0.01 |  |  |  |  |
| HICF0180 | ERS3122729 | Muller Hinton | Staphylococcus aureus | 2,968,170 | 87.82 | 74.44 | 94.2 | 8 | *mecA* |  |  |  |  |  |  |  |  |  |  |  |  |  | 0.01 |  |  |  |  |
| HICF0181 | ERS3122730 | Muller Hinton | Staphylococcus aureus | 3,898,854 | 87.31 | 95.52 | 93.3 | 1 | *mecA* |  |  | 0.06 | 0.03 |  |  | 0.01 |  |  |  |  |  |  | 0.01 |  |  |  |  |
| HICF0182 | ERS3122731 | Muller Hinton | Staphylococcus aureus | 4,039,980 | 86.45 | 99.54 | 93.5 | 1 | *mecA* | 0.01 | 0.01 |  |  |  |  |  |  |  |  | 0.01 |  |  |  |  | 0.02 |  |  |
| HICF0183 | ERS3122732 | Muller Hinton | Staphylococcus aureus | 4,130,040 | 69.92 | 99.48 | 95.2 | 8 | *mecA* | 0.02 | 0.01 | 0.01 | 0.01 |  |  |  |  |  |  |  | 0.02 |  |  |  |  | 0.01 |  |
| HICF0184 | ERS3122733 | Muller Hinton | Staphylococcus aureus | 2,643,696 | 87.41 | 70.17 | 100 | 22 | *mecA* |  |  |  |  |  |  |  |  |  |  |  |  |  |  |  |  |  |  |
| HICF0185 | ERS3122734 | *Brilliance* MRSA | Staphylococcus aureus | 2,542,224 | 85.49 | 61.71 | 95.6 | 36 | *mecA* | 0.01 |  |  |  |  |  |  |  |  | 0.01 |  |  |  | 0.01 |  |  |  |  |
| HICF0186 | ERS3122735 | Muller Hinton | Staphylococcus aureus | 2,151,152 | 87.96 | 56.01 | 99.6 | 22 | *mecA* |  | 0.01 |  |  |  |  |  |  |  |  |  |  |  |  |  |  |  |  |
| HICF0187 | ERS3122736 | CBA/CAP | Staphylococcus aureus | 2,718,412 | 88.86 | 67.85 | 93.4 | 1 | *mecA* | 0.01 |  |  |  |  |  |  |  |  |  | 0.01 |  |  |  |  | 0.02 |  |  |
| HICF0188 | ERS3122737 | Muller Hinton | Staphylococcus aureus | 2,063,934 | 89.08 | 51.63 | 93.1 | 840 | *mecA* | 0.04 |  |  | 0.05 |  |  |  |  |  |  |  |  |  | 0.01 |  |  |  |  |
| HICF0190 | ERS3122738 | Muller Hinton | Staphylococcus aureus | 2,601,548 | 89.19 | 65.02 | 93.1 | 45 | *mecA* |  | 0.01 |  |  |  |  |  |  |  |  |  |  |  |  |  |  |  |  |
| HICF0193 | ERS3122739 | CBA/CAP | Staphylococcus aureus | 2,605,840 | 87.66 | 65.06 | 94 | 30 | *mecA* |  | 0.01 |  |  |  |  |  |  |  |  | 0.51 |  | 0.08 |  |  |  |  |  |
| HICF0194 | ERS3122740 | Muller Hinton | Staphylococcus aureus | 2,690,332 | 80.79 | 64.86 | 99.9 | 22 | *mecA* |  |  | 0.07 | 0.08 |  |  | 0.05 |  |  |  |  |  |  |  |  |  |  | 1.09 |
